# Efficacy of Granulocyte Colony Stimulating Factor in Severe Alcoholic Hepatitis: A Systematic Review and Meta-Analysis

**DOI:** 10.7759/cureus.10474

**Published:** 2020-09-15

**Authors:** Muhammad Baig, Saqib Walayat, Sonu Dhillon, Srinivas Puli

**Affiliations:** 1 Gastroenterology, OSF St. Francis Medical Center/University of Illinois College of Medicine at Peoria, Peoria, USA

**Keywords:** alcoholic hepatitis, granulocyte colony-stimulating factor, systemic steroids

## Abstract

Background

Severe alcoholic hepatitis is a condition with a very high mortality rate and there is a paucity of evidence regarding efficacy and safety of most available therapeutic options. The present systematic review and meta-analysis aims to assess the survival benefit of granulocyte colony stimulating factor (G-CSF) in patients with severe alcoholic hepatitis.

Methods

Studies involving adult patients receiving G-CSF for severe alcoholic hepatitis were searched in MEDLINE, Ovid journals, MEDLINE nonindexed citations, and Cochrane Central Register of Controlled Trials and Database of Systematic Reviews. Pooling was conducted by both fixed and random effects model.

Results

The initial search identified 543 reference articles; of these 24 relevant articles were selected and reviewed. Data was extracted from four studies (*n* = 136) which met the inclusion criteria. In the pooled analysis, the 90-day survival in the G-CSF group was 80.03% (95% CI = 69.93-88.49) compared to 40.92% (95% CI = 29.76-52.58) in the Standard Medical Therapy (SMT) group. At 28 days, the Model for End-Stage Liver Disease (MELD) score lowered by 4.89 (95% CI = 4.13-5.64) in the G-CSF group compared to 4.00 (95% CI = 3.25-4.75) in the SMT group. Child-Turcotte-Pugh score declined by 2.26 (95% CI = 1.90-2.63) in the G-CSF group after 28 days compared to 0.91 (95% CI = 0.59-1.23) in the SMT group. At 28 days, Maddrey Discriminant Function score lowered by 39.79 (95% CI = 34.22-45.36) in the G-CSF group compared to 12.39 (95% CI = 6.90-17.88) in the SMT group.

Conclusions

In patients with severe alcoholic hepatitis, G-CSF therapy resulted in significantly improved 90-day survival compared to SMT. It also demonstrated significant reduction in severity indices (Child-Turcotte-Pugh, MELD, and Maddrey discriminant function) after 28 days of treatment. There certainly is a need for further studies, including development of personalized therapeutic dosing schedules, for G-CSF administration.

## Introduction

Alcoholic hepatitis (AH) has a very high mortality rate, approaching 40% within the first four weeks of clinical presentation [1]. Clinical diagnosis is made in the setting of worsening jaundice in a patient with chronic heavy alcohol use until at least six weeks prior to presentation, elevated liver enzymes, and exclusion of other liver diseases. However, whenever the clinical diagnosis is uncertain, liver biopsy may be needed for further diagnostic clarity [2]. Model of End-Stage Liver Disease (MELD) and Maddrey Discriminant Function (mDF) are commonly used in clinical practice to stratify the severity of an episode of AH and estimate prognosis. MELD scores of >20, mDF scores of >32, or the presence of hepatic encephalopathy indicate severe disease [3,4].

Multiple therapeutic modalities have been investigated for the treatment of such patients. Corticosteroid therapy is the current first-line treatment for severe AH but has remained controversial due to modest reductions in 28-day mortality and no survival benefit beyond one month [5]. Moreover, due to the high incidence of systemic side effects and various absolute and relative contraindications, most treating physicians have to limit their use to only a fraction of these patients [6,7]. The Steroids or Pentoxifylline for Alcoholic Hepatitis (STOPAH) trial also showed no significant morbidity or mortality benefit with pentoxifylline, an alternative therapy for severe AH [8]. Anti-tumor necrosis factor agents, growth factors, and antioxidants were initially deemed promising but later found to be ineffective in further clinical studies as well [1,9]. Literature reveals encouraging results with salvage liver transplantation for patients with severe AH who remain non-responders [10,11,12]. However, stringent inclusion and exclusion criteria limits its utility to only 1 to 2% of cases [10,13]. Amidst all the confusion and unmet need for new therapeutic options, granulocyte colony-stimulating factor (G-CSF) has shown some promise to salvage the remaining patients with severe AH, but the evidence remains limited at best.

G-CSF is an indigenous glycoprotein produced by macrophages, fibroblasts, and endothelial cells which promotes cell growth, differentiation, and function of neutrophils [14]. In human and animal trials, the administration of G-CSF to subjects with liver damage promoted liver repair by increasing the migration of bone marrow progenitors to the liver with clinical, biochemical, and histological improvement [15,16].

There are currently several small reports suggesting benefit of G-CSF therapy in severe AH. The aim of the present meta-analysis was to systematically review the published literature and assess the efficacy of G-CSF therapy in improving survival and liver severity indices in patients with severe AH.

## Materials and methods

Search methodology

A literature search was conducted using the electronic database engines MEDLINE through PubMed, Ovid, Cochrane Library (Cochrane Central Register of Controlled Trials and Cochrane Database of Systematic Reviews), EMBASE, Cumulative Index for Nursing & Allied Health Literature, ACP Journal Club, Database of Abstracts of Reviews of Effects (DARE), International Pharmaceutical Abstracts, OVID Healthstar, and Google Scholar from January 1974 to August 2020 to identify published articles and reports addressing the use of G-CSF in patients with AH. The combinations of keywords used were (“alcoholic hepatitis” or “alcoholic steatohepatitis”) and (“Granulocyte colony-stimulating factor” or “G-CSF”). The reference list of all eligible studies was reviewed to identify additional studies. The retrieved studies were carefully examined to exclude potential duplicates or overlapping data. Titles and abstracts selected from the initial search were first scanned, and the full papers of potential eligible studies were reviewed.

Study eligibility

Published studies were eligible for inclusion if they reported the use of G-CSF for the management of severe alcoholic hepatitis. Articles were excluded if they were not written in English, no outcomes were reported, or they represented review articles or studies published as abstracts only. In studies using multiple modalities for the management of alcoholic hepatitis, data from the cohort of patients who received G-CSF were collected and analyzed. Two reviewers (MB, SP) independently performed study selection according to eligibility criteria. Disagreements were resolved by discussion or a third reviewer. The agreement between reviewers for the collected data gave a Cohen κ value of 1.0.

Data extraction and quality assessment

The following data were independently abstracted onto a standardized form: study characteristics (primary author, time period of study, year of publication, and country of the population studied), study design, baseline characteristics of the study population (the numbers of patients enrolled, participant demographics, biochemical markers, and risk stratification scores when available), the intervention details and outcomes (change in biochemical markers, clinical severity indices, and survival). Risk of bias was rated for each study by two authors independently, using the Cochrane criteria for RCTs [17]. 

Outcome definition

The primary outcome of interest was 90-day survival of severe AH patients receiving G-CSF therapy compared to Standard Medical Therapy (SMT). Effect of G-CSF therapy on severity indices [Child-Turcotte-Pugh (CTP), Model of End-stage Liver Disease (MELD) and Maddrey discriminant function (mDF)] was evaluated as a secondary outcome [18]. 

Statistical analysis

This meta-analysis was performed by calculating pooled proportions. First the individual study proportions were transformed into a quantity using Freeman-Tukey variant of the arcsine square root transformed proportion. The pooled proportion is calculated as the back-transform of the weighted mean of the transformed proportions, using inverse arcsine variance weights for the fixed effects model and DerSimonian-Laird weights for the random effects model [19]. Forest plots were drawn to show the point estimates in each study in relation to the summary pooled estimate. The width of the point estimates in the Forest plots indicates the assigned weight to that study. The heterogeneity among studies was tested using I^2^ statistic and Cochran Q test based upon inverse variance weights [20]. I^2^ of 0% to 39% was considered as nonsignificant heterogeneity, 40% to 75% as moderate heterogeneity, and 76% to 100% as considerable heterogeneity. If P-value is >0.10, it rejects the null hypothesis that the studies are heterogeneous. The effect of publication and selection bias on the summary estimates was tested by Harbord-Egger bias indicator [21].

## Results

**Figure 1:**
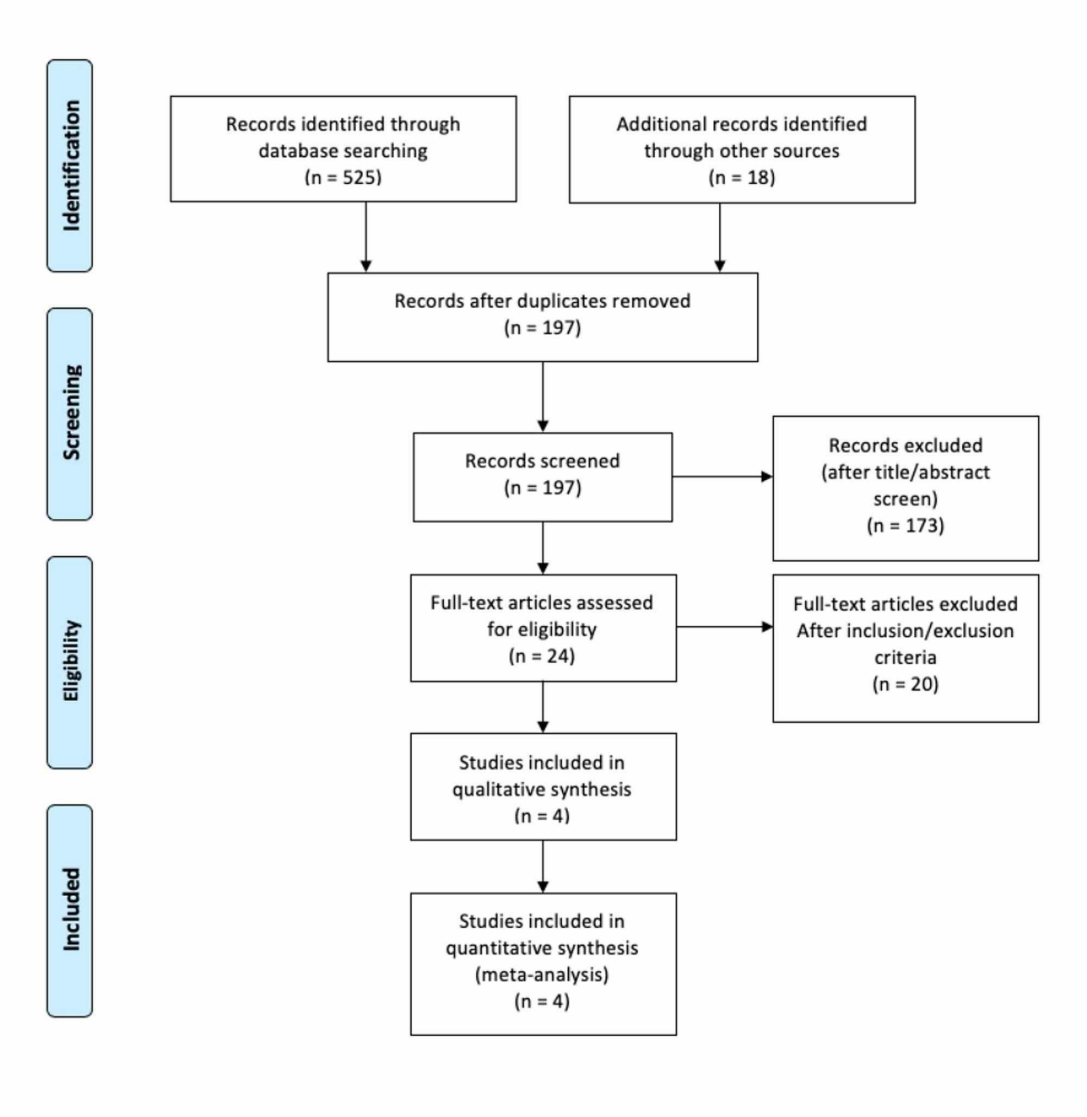
Preferred Reporting Items for Systematic Reviews and Meta-Analyses flow diagram detailing the review process.

The initial search identified 543 reference articles, of which 24 articles were selected and reviewed. Data were extracted from four studies that evaluated the efficacy of G-CSF in severe alcoholic hepatitis, which met the inclusion criterion [22,23,24,25]. A Preferred Reporting Items for Systematic Reviews and Meta-Analyses (PRISMA) flow diagram for details of the review process is shown in Figure 1 [26]. All included studies were randomized controlled trials and published in English as full-text articles. Pooled estimates were calculated by the fixed effect model for better accuracy based on the nature of individual study characteristics and heterogeneity. The P for Chi-squared heterogeneity for all the pooled accuracy estimates was >.10.

The total number of patients included in this meta-analysis was 136. Mean age of the patients in the G-CSF group was 43.44 years compared to 45.32 years in the SMT group. Three studies used G-CSF dose of 10 mcg/kg for five consecutive days [23,24,25]. One trial used 5 mcg/kg daily for five days followed by every third day until four weeks [22]. Table 1 shows the baseline characteristics of the studies. 

**Table 1:** Basic characteristics of the included studies. N = total number of patients in each study, N-GCSF = number of patients in each study receiving granulocyte colony-stimulating factor (GCSF), N-Placebo = number of patients in each study receiving placebo with standard of care, RCT = randomized controlled trial.

No.	Study	Year	Type	N	N-GCSF	N-Placebo
1	Shasthry et al [22]	2019	RCT	28	14	14
2	Singh et al [23]	2014	RCT	46	23	23
3	Singh et al [24]	2018	RCT	38	18	20
4	Spahr et al [25]	2008	RCT	24	13	11

Primary outcome (90-day survival)

**Figure 2:**
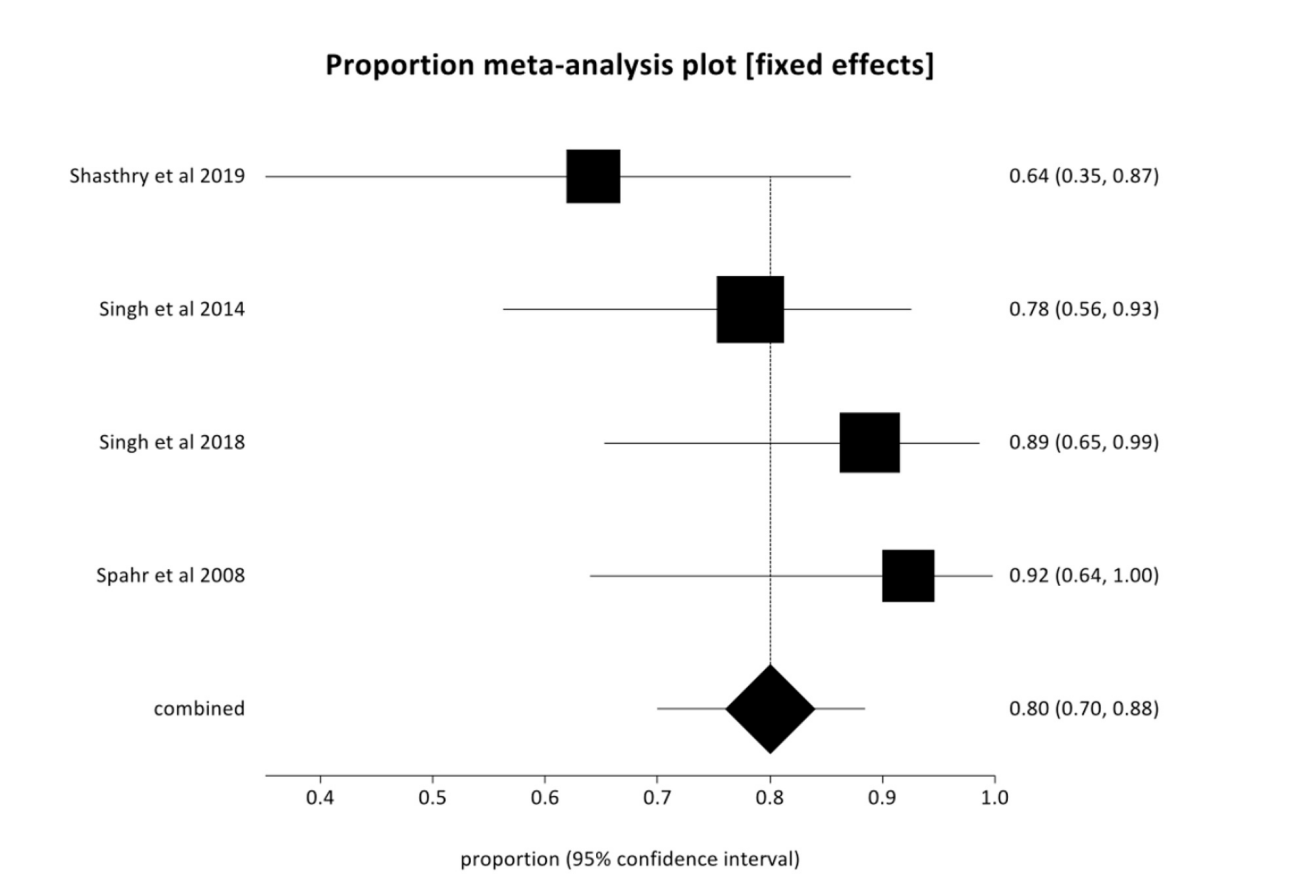
Forest plot—Individual study proportions and the pooled estimate of 90-day survival with granulocyte colony stimulating factor (G-CSF) therapy in severe alcoholic hepatitis (AH) (fixed effects).

**Figure 3:**
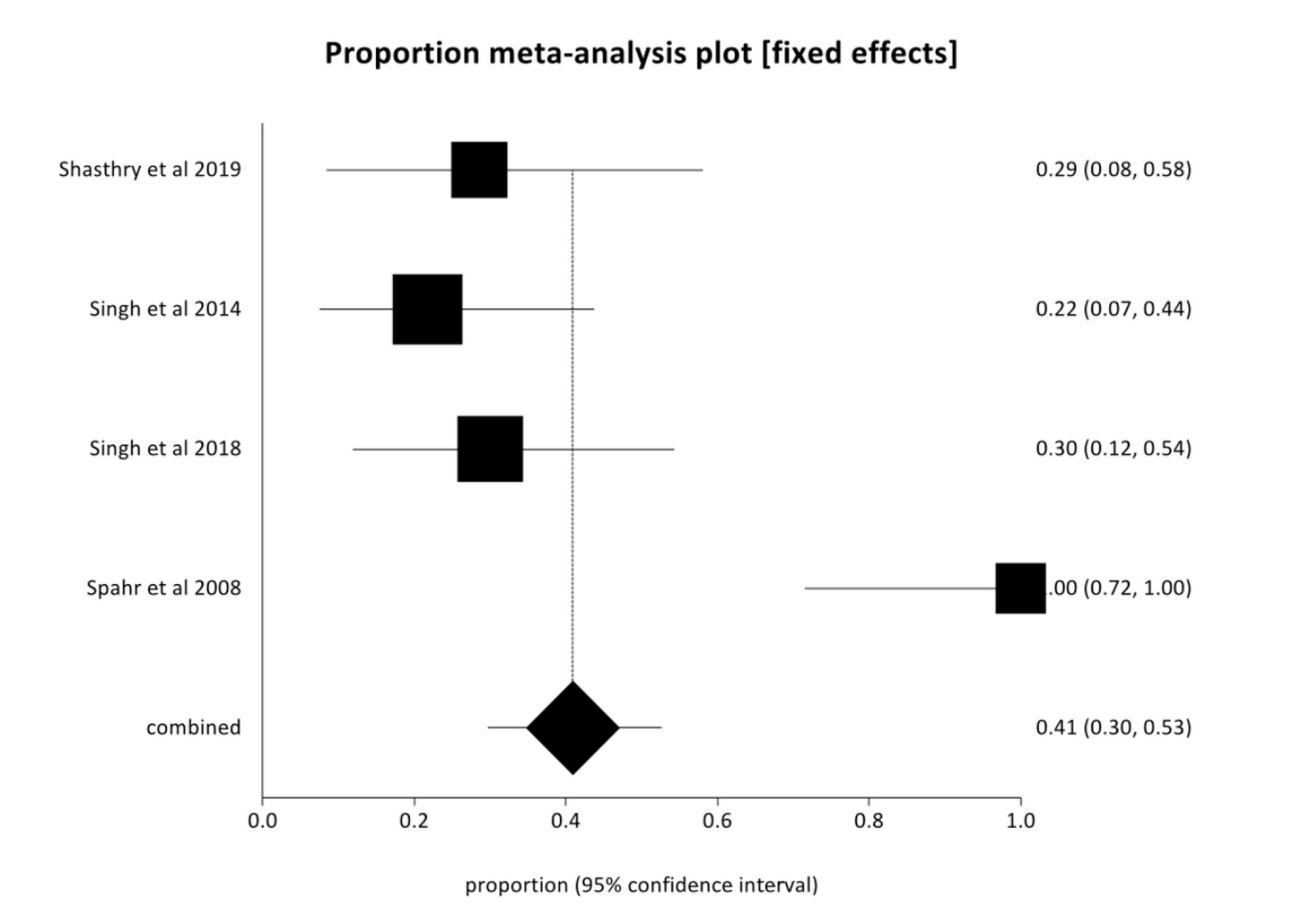
Forest plot—Individual study proportions and the pooled estimate of 90-day survival with placebo and standard of care therapy in severe alcoholic hepatitis (AH) (fixed effects).

In the pooled analysis, 90-day survival in the G-CSF group was 80.03% (95% CI  = 69.93-88.49) compared to 40.92% (95% CI  = 29.76-52.58) in the SMT group with low heterogeneity (I^2^ = 20.3%). Figures 2, 3 are forest plots representing the pooled and individual 90-day survival rates with G-CSF therapy and SMT, respectively. Publication bias calculated using Harbord-Egger bias indicator gave a value of -5.23 (95% CI = -15.35-4.9, P = 0.16), indicating no publication bias. 

Secondary outcomes

MELD Score

At 28 days, G-CSF therapy lowered the MELD score by 4.89 (95% CI = 4.13-5.64) compared to a reduction of 4.00 (95% CI = 3.25-4.75) in the SMT group. Moreover, at 90 days G-CSF therapy further reduced the MELD score by 11.94 (95% CI = 11.04-12.84) compared to baseline. 

Child-Turcotte-Pugh Score

At 28 days, G-CSF therapy lowered the CTP score by 2.26 (95% CI = 1.90-2.63) which was significantly better than a reduction of 0.91 (95% CI = 0.59-1.23) in the SMT group. Moreover, at 90 days G-CSF therapy further lowered the CTP score by 3.21 (95% CI = 2.85-3.57) compared to baseline. 

Maddrey Discriminant Function Score

At 28 days, G-CSF therapy lowered the mDF score by 39.79 (95% CI = 34.22-45.36) which was significantly better than a reduction of 12.39 (95% CI = 6.90-17.88) in the SMT group. Moreover, at 90 days G-CSF therapy further lowered the mDF score by 65.07 (95% CI = 60.16-69.97) compared to baseline. 

## Discussion

Severe alcoholic hepatitis remains a commonly encountered clinical entity with high degree of morbidity and mortality and no satisfactory therapeutic option. Corticosteroid therapy remains the standard of medical care but literature supports only modest reductions in 28-day mortality with no long-term survival benefit [5]. High incidence of systemic side effects and risk of infectious complications likely contribute to lack of significant mortality reduction [6,7]. Studies on pentoxifylline, anti-TNF agents, growth factors, and antioxidants also failed to demonstrate any survival benefit in this high-risk patient population [1,8,9].

Recombinant G-CSF, first approved in 1991 to reduce the incidence of neutropenic fever after myelosuppressive chemotherapy, causes proliferation and differentiation of bone marrow precursor cells into mature granulocytes [27]. Further studies have suggested its potential role in differentiation of bone marrow precursor cells into hepatocytes promoting liver regeneration [15,16,25]. The evidence regarding the latest is not universally conclusive, since some studies have been unable to reproduce these findings [28,29]. However, the effects of G-CSF in liver regeneration are not limited to the mobilization and differentiation of bone marrow-derived stem cells. G-CSF also exerts autocrine and paracrine effects in the liver, promoting and enhancing the oval cell reaction [15]. The synergistic contribution of the bone marrow-derived stem cells and the oval cells might be responsible for the liver function improvement observed in the cited studies. Spahr et al. first demonstrated the efficacy of G-CSF therapy in patients with alcoholic hepatitis, liver failure, and cirrhosis [25]. Chavez-Tapia et al. have also reported short-term mortality reduction and improvement in liver function with G-CSF therapy specifically in acute on chronic liver disease patients [30]. However, the evidence supporting G-CSF therapy to salvage patients with AH still remains limited at best.

Several case reports and small studies have investigated the use of G-CSF in severe AH. To the best of our knowledge, this is the first systematic review and meta-analysis to assess its role in improving survival and liver severity indices of patients with severe AH. Patients treated with G-CSF were noted to have significantly improved 90-day survival compared with standard medical therapy (80.03% versus 40.92%). This significant survival benefit is likely due to the role of G-CSF therapy in liver regeneration with reduced incidence of infectious complications compared to corticosteroids [22]. As the mobilization of hematopoietic stem cells occurs slowly over time, the survival benefit of G-CSF therapy is also long-term as seen in the prior literature and confirmed by our meta-analysis [16,23,25]. 

All included studies and their pooled analysis provided evidence of improved liver function demonstrated by significant reduction in CTP, MELD, and mDF scores at 28 days. The robust decline of mDF score by 39.79 in the G-CSF group compared to 12.39 in the SMT group is reassuring. All included studies reported continued improvement of liver function with G-CSF therapy even at 90 days. No significant adverse events were reported in any of the trials. Deaths reported among patients receiving G-CSF therapy were owing to progressive liver failure with no mortality reported directly as a complication of therapy [24]. As expected, there was an increase in neutrophil count with G-CSF therapy and only two patients required dose reduction due to significant leukocytosis (over 40,000/cm^3^) [23]. Singh et al. reported significant increase in spleen size with G-CSF therapy but no patient progressed to develop splenic rupture [24]. Minor bone pains were reported in two studies and only one patient reported by Shastry et al. required a reduction in the frequency and number of G-CSF doses to counter this side effect [22,25].

Our meta-analysis, however, has some important limitations. The number of trials included in this review is small and restricted to South Asian (India) and European populations, limiting the external validity of the results [22,23,24,25]. It is possible that the diagnosis of AH might have been incorrect in a small number of the patients due to absence of diagnostic confirmation with liver biopsy. The majority of the included trials did not report important information such as number of patients with underlying chronic liver disease, number of patients undergoing liver transplantation, and length of hospitalization, precluding the analysis of these important outcomes. Due to limited 90-day survival in the placebo arm, severity indices at 90 days were not reported in most of the studies and hence not included in our analysis. 

## Conclusions

In summary, G-CSF could be an additional rescue therapeutic option in patients with severe AH. It demonstrated a significant improvement of liver function and reduction in 90-day mortality with no significant adverse events. Based on the encouraging outcomes reported in the literature and our meta-analysis along with a long safety record of use in cancer patients, further randomized controlled trials evaluating the effectiveness of G-CSF in patients with alcoholic hepatitis are required.
